# Polymorphism and structure formation in copper phthalocyanine thin films

**DOI:** 10.1107/S1600576720015472

**Published:** 2021-02-01

**Authors:** Berthold Reisz, Valentina Belova, Giuliano Duva, Clemens Zeiser, Martin Hodas, Jakub Hagara, Peter Šiffalovič, Linus Pithan, Takuya Hosokai, Alexander Hinderhofer, Alexander Gerlach, Frank Schreiber

**Affiliations:** a Institute for Applied Physics, University of Tübingen, Auf der Morgenstelle 10, 72076 Tübingen, Germany; b Institute of Physics, Slovak Academy of Sciences, Dúbravská cesta 9, 845 11 Bratislava 45, Slovak Republic; c European Synchrotron Radiation Facility, 71 avenue des Martyrs CS 402200, 38043 Grenoble Cedex 9, France; d National Metrology Institute of Japan (NMIJ), National Institute of Advanced Industrial Science and Technology (AIST), Tsukuba Central 5, 1-1-1 Azuma, Tsukuba, Ibaraki 305-8565, Japan

**Keywords:** polymorphism, organic semiconductors, thin films, atomic force microscopy, morphology, reciprocal space mapping, X-ray reflectivity, grazing-incidence X-ray diffraction, *GenX*

## Abstract

Many polymorphic crystal structures of copper phthalocyanine (CuPc) have been reported over the past few decades, but despite its manifold applicability, the structure of the frequently mentioned α polymorph remained unclear. The base-centered unit cell (space group *C*2/*c*) suggested in 1966 was ruled out in 2003 and was replaced by a primitive triclinic unit cell (space group *P* 1). This study proves unequivocally that both α structures coexist in vacuum-deposited CuPc thin films on native silicon oxide by reciprocal space mapping using synchrotron radiation in grazing incidence. The unit-cell parameters and the space group were determined by kinematic scattering theory and provide possible molecular arrangements within the unit cell of the *C*2/*c* structure by excluded-volume considerations. *In situ* X-ray diffraction experiments and *ex situ* atomic force microscopy complement the experimental data further and provide insight into the formation of a smooth thin film by a temperature-driven downward diffusion of CuPc molecules during growth.

## Abstract 2

This X-ray diffraction study proves that two α polymorphs of copper pthalocyanine (CuPc) co-exist in vacuum-deposited thin films and provides possible molecular configurations by excluded-volume considerations. Furthermore, atomic force microscopy images together with a simple MATLAB simulation show that elevated substrate temperatures facilitate the downward diffusion of CuPc molecules during film growth and lead to a smoother surface.

## 1. Introduction   

Together with the possibility of tuning growth parameters such as substrate temperature and deposition rate during organic molecular beam deposition (Forrest, 1997 ▸), the application of organic compounds in electronic devices has attracted increasing attention (Witte & Wöll, 2004 ▸). Copper phthalocyanine (CuPc) is an important compound for application in electronic and optoelectronic devices and serves as a model for the entire family of phthalocyanines. Examples of technical applications of CuPc are organic solar cells (Tang, 1986 ▸), light emitting diodes (Hill & Kahn, 1999 ▸; Riel *et al.*, 2001 ▸), transistors (Bao *et al.*, 1996 ▸) and gas sensors (Berger *et al.*, 2000 ▸).

The functionality and efficiency of these devices crucially depend on their thin-film structure (Opitz *et al.*, 2010 ▸) and the molecular orientation of CuPc, which has been investigated several times (Suito *et al.*, 1962 ▸; Nonaka *et al.*, 1995 ▸; Nakamura *et al.*, 1996 ▸; Hiesgen *et al.*, 2000 ▸; Berger *et al.*, 2000 ▸). Despite its importance, the occurring crystal structures of CuPc are not yet fully resolved.

In 1935, Robertson had already prepared needle-like single crystals of CuPc by low-pressure sublimation and determined the unit-cell parameters, the space group *P*2_1_/*a* and the molecular orientation by X-ray diffraction (Robertson, 1935 ▸). Initially, CuPc was mainly used as a commercial dye in the form of either a powder or a paste, although different color shades appeared. Hamm & Van Norman (1948 ▸) suggested that the color variations stem from different CuPc crystal structures rather than crystal size, as had been assumed until then.

With the collection of X-ray diffraction rings for the recognition of a set of standard dyes, three polymorphic structures of CuPc were reported, namely the α, the β and the γ forms (Susich, 1950 ▸). The α and β forms were identified as different polymorphs owing to clearly different X-ray diffraction patterns and different infrared absorption spectra. The stable β polymorph was assigned to the crystal structure determined by Robertson, but the crystal structure of the less stable α polymorph remained unknown (Ebert & Gottlieb, 1952 ▸). Although the preparation of a γ polymorph of CuPc was reported (Eastes, 1956 ▸), infrared spectroscopy revealed some years later that these crystals were in fact different kinds of α polymorphs (Assour, 1965 ▸). The first attempt to determine the crystal structure of the α polymorph was made by Robinson & Klein (1952 ▸), who suggested a tetragonal unit cell, probable space group *P*4/*m*. The α polymorph was obtained via the thus-far conventional method from the dried CuPc precipitate of a sulfuric acid solution. In the following years, direct sublimation onto a substrate under low pressure was found to be an alternative way to produce small α-CuPc crystals (Karasek & Decius, 1952 ▸). It was shown that the crystals were highly oriented with respect to the substrate (Suito *et al.*, 1962 ▸; Nakamura *et al.*, 1996 ▸).

Finally, Honigmann *et al.* (1965 ▸) and Ashida *et al.* (1966 ▸) delivered a crystal structure for the α form. A base-centered monoclinic α structure (*C*2/*c*) containing four molecules per unit cell was found by electron diffraction on CuPc deposited under vacuum at 423 K on mica and on potassium chloride (Ashida *et al.*, 1966 ▸). The crystal structures of α-CuPc and β-CuPc were redetermined for deposition with the Cambridge Crystallographic Data Centre. The initial β structure of CuPc found was confirmed by Brown (1968 ▸); however, the diffraction pattern of the α structure could not be reproduced (Hoshino *et al.*, 2003 ▸). For this reason, the α structure (*C*2/*c*) from 1966 was ruled out and a less symmetrical unit cell (space group *P* 1) was suggested instead by Hoshino *et al.* (2003 ▸).

The present study confirms that both α structures of CuPc, the *C*2/*c* and the *P* 1 polymorphs, coexist. A continuous transition from the less stable α structure to the more stable β structure with several intermediate states during post-growth annealing was reported (Heutz *et al.*, 2000 ▸; Berger *et al.*, 2000 ▸), which accounts for the large number of reported polymorphs. We assume that, in addition to the coexistence of different polymorphs, the molecular arrangement of the unit cell also differs from crystallite to crystallite in the 2D powder of the CuPc thin films, which might be the reason why the calculation of diffracted peak intensities failed in prior studies. We provide excluded-volume considerations for the structure determination instead and complement our results by investigation of the influence of substrate temperatures during the CuPc film growth.

## 2. Experimental   

**Figure 1:**
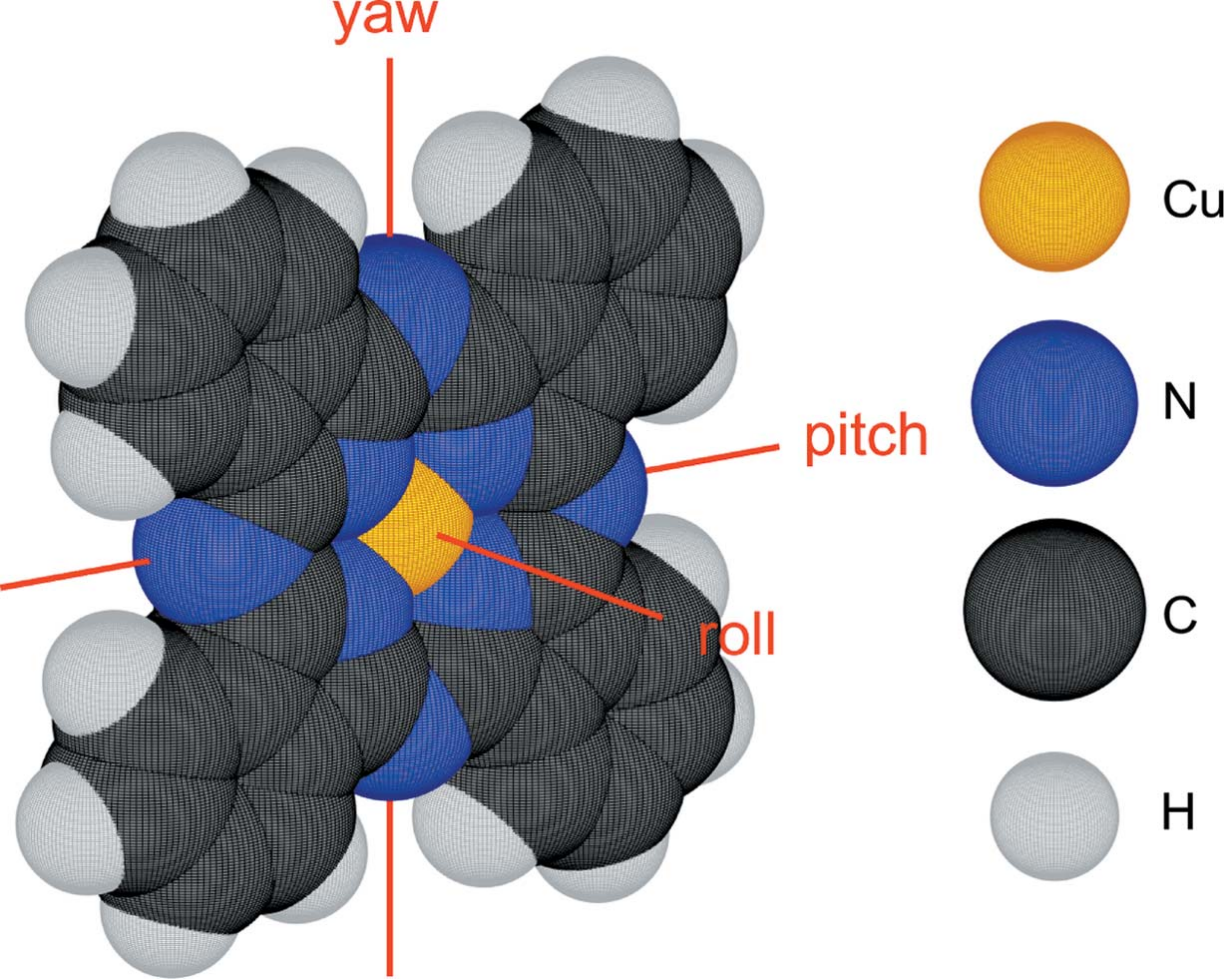
Chemical structure of copper phthalocyanine (CuN_8_C_32_H_16_, CuPc) and the size of the van der Waals radii: copper (1.40 Å), nitrogen (1.55 Å), carbon (1.70 Å) and hydrogen (1.20 Å) as reported by Bondi (1964).

Copper phthalocyanine (Fig. 1 ▸) was purchased from Sigma–Aldrich (purity 99.9% by gradient sublimation) and installed in a portable vacuum chamber equipped with a 360° beryllium window for *in situ* X-ray diffraction experiments during and after organic molecular beam deposition (Ritley *et al.*, 2001 ▸). A 20 × 10 mm silicon substrate covered with a native amorphous oxide layer (∼1–2 nm thick) was cut from a standard {100} p-type silicon wafer, cleaned with acetone and 2-propanol in an ultrasonic bath for 5 min each, and then heated under vacuum above 500 K for 10 h.

The molecules were evaporated from a Knudsen effusion cell and deposited onto the substrate at two different substrate temperatures, 310 and 400 K, in order to investigate the influence of the substrate temperature on film growth. A deposition rate of 2.0 Å min^−1^ was maintained and monitored by a quartz crystal micro-balance for 100 min at an average pressure of approximately 1 × 10^−9^ mbar during the entire growth, resulting in an average film thickness of 200 Å.

X-ray reflectivity (XRR) and grazing incidence X-ray diffraction (GIXD) experiments were carried out *in situ* directly after the deposition at the materials science beamline MS-X04SA of the Swiss Light Source (Willmott *et al.*, 2013 ▸) at a beam energy of 12.7 keV. The angle of grazing incidence (0.120°, 0.027 Å^−1^) was chosen to be close to the total reflection edge of silicon, probing the crystal structure throughout the entire film thickness at maximum signal.

Another two samples were prepared in our home laboratory under the same conditions, *i.e.* the same base pressure, substrate temperatures and deposition rate (2.0 Å min^−1^), resulting in the same average film thickness of 200 Å after 100 min of deposition. The samples were kept under vacuum until the substrate cooled to room temperature. After the samples had been transferred to air, they were examined *ex situ* by atomic force microscopy (AFM) using a JPK Nanowizard II instrument operating in tapping mode. AFM images of each sample, 3 × 3 µm in size, were acquired.

Finally, reciprocal space maps (*Q* maps) for both samples were measured and GIXD profiles of both samples were obtained *ex situ* at the ID03 beamline of the European Synchrotron Radiation Facility (ESRF) using a 2D-Maxipix detector. The X-ray energy was set to 24.0 keV and the angle of incidence (0.030°, 0.013 Å^−1^) was set below half the total reflection angle of silicon, probing mainly the surface.

## 3. Analysis   

### 3.1. Crystal structure determination   

First, the unit cell and its orientation with respect to the substrate surface were determined from the peak positions in the *Q* map. In a second step, the space group was determined by comparing the Miller indices (*hkl*) of extinct Bragg peaks with the reflection conditions for each space group listed in *International Tables for Crystallography*, Vol. A (Hahn & Looijenga-Voss, 2006 ▸). The same procedure was applied to the GIXD patterns. Furthermore, the size of coherently scattering crystallites *d*_coh_ along the *c* axis (see Table 1 ▸) was determined from the full width at half-maximum Δ*q* of the (00±1) GIXD peak using the Scherrer formula *d*_coh_ ≃ 2π/Δ*q*. In a third step, possible molecular orientations were determined by excluded-volume considerations, and the corresponding peak intensities were calculated and compared with the experimental data. All calculations were carried out with the help of several custom-written MATLAB scripts.

### 3.2. Determination of the molecular arrangement within the unit cell   

An upright standing molecule aligned with the unit-cell axis was chosen as the starting configuration. The yaw axis was aligned perpendicular to the *bc* plane, the roll axis parallel to the short *b* axis and the pitch axis parallel to the *c* axis (Fig. 1 ▸). Every configuration was obtained from this starting configuration by rotating the molecule systematically around each of its symmetry axes (yaw, pitch and roll) in steps of 1° from −45 to +45°. Keeping the order of rotation, first the yaw, then the pitch and finally the roll axis, assures an unequivocal definition. The yaw angle defines the in-plane orientation with respect to the unit-cell axes. The pitch angle defines the molecular tilt with respect to the substrate surface and the roll angle defines the rotation around the unique fourfold symmetry axis of CuPc. All other molecules within the same unit cell were rotated synchronously to the first by applying the symmetry operations of the present space group *C*2/*c*, taken in terms of equivalent Wyckoff positions from the Bilbao Crystallographic Server (Aroyo *et al.*, 2006 ▸).

### 3.3. Excluded-volume considerations   

Due to the limited space within the unit cell, only a finite number of configurations can be physically reasonable. The overlap between neighboring molecules was calculated for each configuration, considering the van der Waals radii of carbon (1.70 Å), nitrogen (1.55 Å), hydrogen (1.20 Å) and copper (1.40 Å) reported by Bondi (1964 ▸). In addition to overlaps inside the unit cell, overlaps between molecules from neighboring unit cells were taken into account by applying periodic boundary conditions. The degree of overlap was determined for each pair of atoms by calculating their distance and subtracting it from the sum of their van der Waals radii. Atomic pairs that were further away from each other than the sum of their van der Waals radii and atoms belonging to the same molecule were ignored. The sum of the remaining pairs of atoms finally delivers the total degree of overlap for each configuration. Diffraction peak intensities for some configurations were calculated using kinematic scattering theory based on the atomic scattering factors taken from *International Tables for Crystallography*, Vol. C (Maslen *et al.*, 2006 ▸).

### 3.4. Layer coverages and roughness from *in situ* XRR   

The XRR profiles were evaluated using the software *GenX*, which is based on the Parratt formalism and is able to simulate X-ray and neutron reflectivity curves for user-defined thin-film models consisting of several layers (Björck & Andersson, 2007 ▸). The thin-film model for this study was built up as follows: starting with a 575 µm-thick silicon substrate and a thin silicon oxide layer on top.

The electron density of amorphous silicon oxide (0.66 e Å^−3^) and the electron density of pure silicon (0.70 e Å^−3^) were calculated from their mass densities (amorphous SiO_2_: ρ = 2.2 g cm^−3^; pure Si: ρ = 2.3 g cm^−3^) as reported by Hofmann *et al.* (1973 ▸). A void layer between the organic thin film and the substrate simulates a possible depletion of charges at the film–substrate interface.

**Figure 2:**
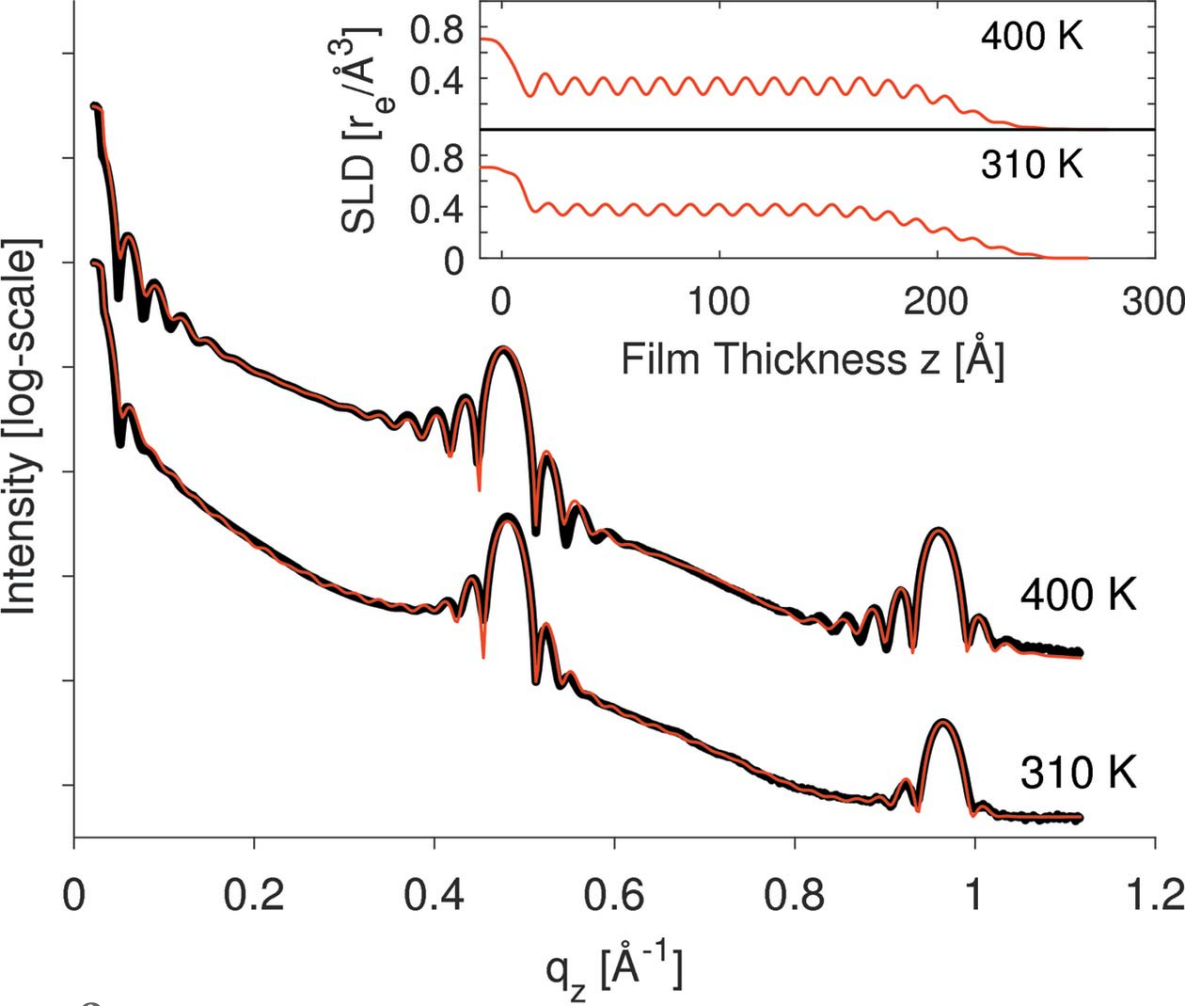
*In situ* X-ray reflectivity (XRR) profiles: experimental data (black line), simulated data (red line) and corresponding SLD of electrons.

The organic thin film itself is made up of several bilayers. Each bilayer consists of a CuPc layer and a void layer simulating the periodically varying electron density in the vertical direction from the bottom to the top, which is shown as the scattering length density (SLD) in the upper right corner of Fig. 2 ▸. The bilayer thickness (∼13.1 Å) was determined from the position of the XRR Bragg peaks and assumed to be the same for all layers. Partially filled layers were simulated by lowering their electron density proportional to their layer coverages Θ_*n*_. Missing values for thickness, roughness and electron density of the individual layers were fitted by the software *GenX* until the experimental (black) and simulated curves (red) agreed (Fig. 2 ▸). Agreement, in the context of *GenX*, means that the figure of merit (FOM), which is a measure of the deviation between the experimental and simulated curves, approaches a constant value below 0.1 (Björck & Andersson, 2007 ▸). Details about the layer model and the fitting procedure can be found in Fig. S1 of the supporting information. Finally, the root mean square roughness σ_RMS_ was estimated from the damping of Kiessig oscillations in the low-*q_z_* range up to 0.2 Å^−1^ (Fig. 2 ▸).

### 3.5. Layer coverages and roughness from *ex situ* AFM   

All heights measured by AFM were divided into 100 classes from zero up to the maximum height and their occurrences were calculated as percentages. The distribution of heights is shown as histograms on the left side of each AFM image (see Fig. 5). The root mean square roughness σ_RMS_ was directly extracted from the distribution of heights and the coverage Θ_*n*_ of each layer was determined by a vertical scan through the AFM profiles in steps of 13.1 Å from bottom to top. Note that the lowest data points in the AFM images are not necessarily the substrate level, since there might be further completely filled layers below. The number of completely filled layers was chosen such that the total amount of deposited material approaches the values determined from XRR. Moreover, the number of islands per area was automatically counted by a custom-written MATLAB program and is reported as island density ρ in Table 1 ▸.

### 3.6. Layer coverages and roughness from simulation   

For comparison of how the layer coverage would look if there was neither diffusion nor any kind of interaction (see Fig. 6), a random deposition of 15 × 3000^2^ particles on a 3000 × 3000 lattice was simulated by a simple MATLAB script. The number *N* = 15 was chosen for our study such that the amount of deposited material per area is 15 × 13.1 Å = 196.5 Å, in agreement with the experiment (2 Å min^−1^ × 100 min = 200 Å). It is well known that the heights resulting from a random deposition of non-diffusing particles are Poisson distributed and that σ_RMS_ is proportional to *N* ^1/2^ for an average height of *N* layers (Chopra, 1969 ▸; Krug, 2002 ▸). Several similar but more complex simulations were carried out in previous work confirming this result (Cerofolini, 1975 ▸; Family & Vicsek, 1985 ▸; Wolf & Villain, 1990 ▸).

## 4. Results   

### 4.1. Crystal structure   

**Figure 3:**
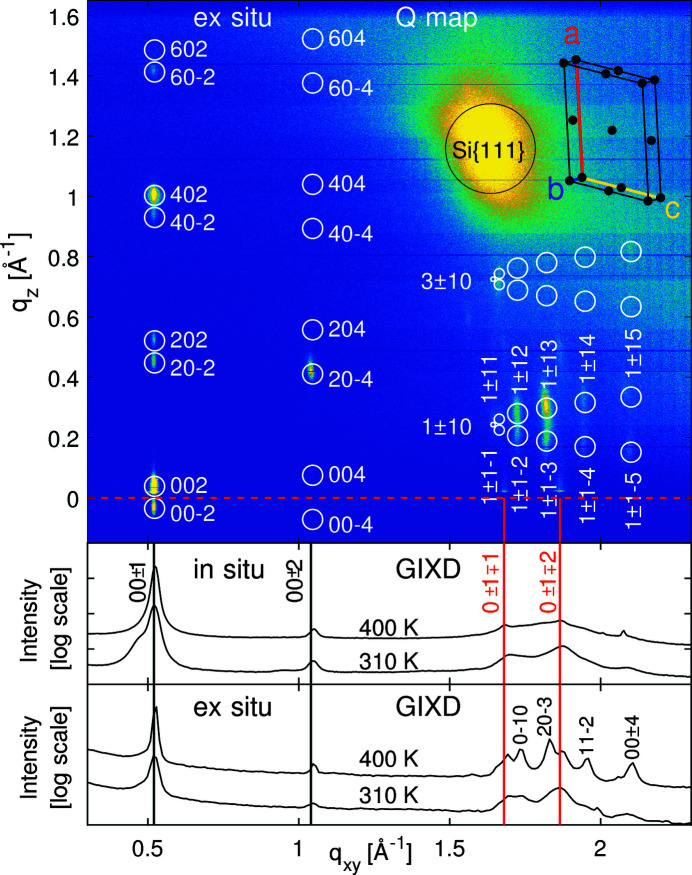
Reciprocal space map (*Q* map) of CuPc grown at 400 K on native silicon oxide (2 Å min^−1^ for 100 min). White circles indicate the calculated peak positions for the *C*2/*c* structure. The corresponding unit cell is shown in the upper right corner [*a* = 26.(1), *b* = 3.8(2), *c* = 24.(0) Å, β = 94.(0)°]. Black dots mark the positions at which the molecules have to be placed in consideration of the space-group symmetries. The GIXD patterns are indexed below according to the primitive triclinic crystal structure (*P* 1) reported by Hoshino (2003), whose* a* and *c* axes are halved.

Fig. 3 ▸ shows the *Q* map of CuPc grown at 400 K. The *Q* map of CuPc grown at 310 K is shown in Fig. S2 of the supporting information for comparison. Peak positions and relative peak intensities agree at both substrate temperatures, although the peaks are markedly stronger at 400 K.

First, the unit-cell parameters [*a* = 26.(1), *b* = 3.8(2), *c* = 24.(0) Å, α = 90, β = 94.(0), γ = 90°] were determined from the peak positions. We obtained a precision of two significant figures from this *Q* map for all unit-cell lengths. The uncertainty of the third figure is indicated by parentheses and comes from the large peak widths and the weaker signal at 310 K. The short *b* axis can be determined accurately with at least one decimal place, which can be attributed to the smaller peak widths in the *q_xy_* direction and the inverse lengths in reciprocal space. Adding 0.1 Å to the length of a short unit cell leads to a much larger shift of peak positions in reciprocal space than adding 0.1 Å to the length of a long unit-cell vector. The unit-cell lengths are closest to the α structure (*C*2/*c*) reported by Ashida *et al.* (1966 ▸) and deviate by less than 1 Å from our values.

The angle β had to be changed from 90.4 to 94.0 ± 0.8°, which is responsible for the vertical splitting into (*h* *k*+*l*) and (*h* *k*−*l*) reflections. The variation of ±0.8° is caused by the vertical elongation of the diffraction peaks. To which degree the reported unit-cell dimensions deviate from each other most likely depends on the choice of substrate, which was mica and potassium chloride in 1966 and native silicon oxide for this study. For a comparison of reported unit cells and crystal structures of CuPc see Table S1 of the supporting information. Despite the large differences between the unit-cell dimensions, the characteristic peak extinctions confirm that the space group must be *C*2/*c*. Only (*h*0*l*) reflections with *h* and *l* being even and (*h*±1*l*) reflections with *h* being odd remain visible, and these are indicated in Fig. 3 ▸ by white circles.

All other peaks in between are extinct due to the *C* centering in the *ab* plane, the twofold screw axis along the *b* direction and the gliding mirror plane along the *c* direction. Peaks with large Miller indices are not visible, presumably due to the limited crystallite sizes. In order to avoid an overcrowded figure, only (*h*±1*l*) peaks for *h* = 1 are labeled and the peaks for *h* = 3 are indicated without labeling. The peaks for *h* = 5 are covered by the strong silicon {111} reflection stemming from the substrate.

Last but not least, two weak peaks appear between *q_xy_* = 1.5 Å^−1^ and *q_xy_* = 2.0 Å^−1^, slightly above the red dotted line at *q_z_* = 0 Å^−1^. They cannot be explained by the space group *C*2/*c*, but agree with the primitive triclinic unit cell (space group *P* 1) reported by Hoshino *et al.* (2003 ▸), whose *a* and *c* axes are halved.

On the other hand this primitive structure cannot explain most of the other peaks seen in the reciprocal space map. So we infer from the *Q* map that both α structures, *C*2/*c* and *P* 1, coexist. Peaks of both α structures also appear in the GIXD patterns. Fig. 3 ▸ presents *in situ* GIXD profiles of the first set of samples and *ex situ* GIXD profiles of the second set of samples. In both data sets, the (00±1) peak of the *P* 1 structure is superimposed by the huge (00±2) peak of the *C*2/*c* structure. The remaining GIXD peaks stem solely from the primitive structure *P* 1. They are substantially smaller for the *in situ* samples, indicating that only a small fraction of the crystallites exhibit the primitive structure *P* 1, particularly when considering that the intensity scale is logarithmic. The *ex situ* GIXD profiles exhibit much taller and sharper *P* 1 peaks, indicating that the *P* 1 crystallites become larger and more abundant at the surface. We recall that the *in situ* data were measured at an incidence angle close to the total reflection of silicon, whereas the *ex situ* data were acquired at half the total reflection angle of silicon, which is a more surface-sensitive measurement. The differences between *in situ* and *ex situ* data should not be over-interpreted. A previous study has shown that the crystallites in organic thin films grow larger at the surface than at the bottom during organic molecular beam deposition (Banerjee *et al.*, 2013 ▸).

### 4.2. Unit-cell orientation   

First we discuss the orientation of the unit cell with respect to the substrate surface and secondly the orientation of the molecules within the unit cell. Regarding the peak positions in the *Q* map (Fig. 3 ▸), the *b* and *c* axes are parallel to the substrate surface. The *a* axis (26.1 Å) is twice the length of the vertical lattice spacing (∼13.1 Å) determined from XRR such that two molecules can be stacked on top of each other within each unit cell. Also the *c* axis (24.0 Å) provides enough space for two molecules. Four molecules per unit cell stacked in rows along the short *b* axis and hence also along the substrate surface result in good agreement with previous studies (Suito *et al.*, 1962 ▸; Nakamura *et al.*, 1996 ▸). Note the shoulder at the left side of the first GIXD peak at 310 K in Fig 3 ▸, which indicates that the unit cell of some crystallites is tilted around the short *b* axis by 86° such that the *ab* plane instead of the *bc* plane is parallel to the substrate surface. On the basis of the knowledge that the *a* axis (26.1 Å) is longer than the *c* axis (24.0 Å), it is clear that this shoulder has to appear on the left side in reciprocal space. Interestingly, this shoulder appears only at 310 K but not at 400 K. We speculate that the partial unit-cell rotation at 310 K is a kinetic effect induced by the growth process. At higher substrate temperatures such kinetic effects are less likely to appear since the diffusion length of the molecules is larger.

### 4.3. Molecular arrangement within the unit cell   

**Figure 4:**
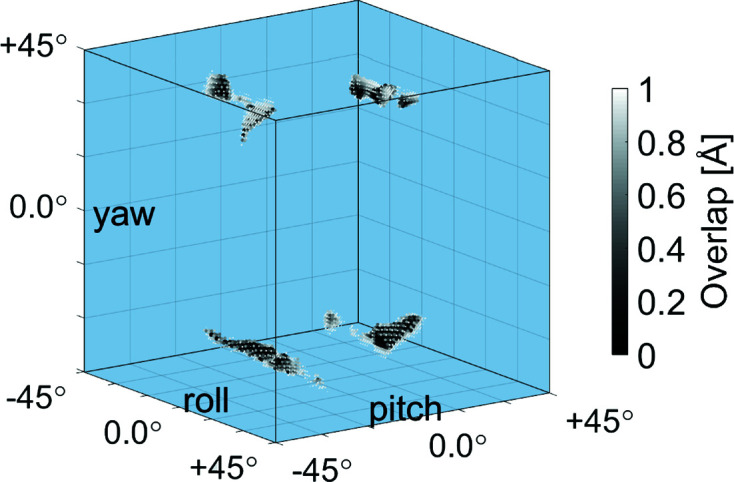
Configuration space: the molecules were systematically rotated within the unit cell (*a* = 26.1, *b* = 3.82, *c* = 24.0 Å, β = 94.0°, space group *C*2/*c*) around their symmetry axes (yaw, pitch and roll) in steps of 1° from −45 to +45°. Configurations with a total degree of overlap between neighboring molecules of less than 1.0 Å are shown as dark spots in this visualization of the resulting 91 × 91 × 91 matrix.

In order to determine possible molecular orientations within the unit cell, the molecules were systematically rotated around their yaw, pitch and roll axes (Fig. 1 ▸). The overlap with neighboring molecules was calculated as described in Section 3 and visualized in a 91 × 91 × 91 matrix (Fig. 4 ▸). It turned out that many *C*2/*c* configurations without overlap are possible. However, none of them exhibit a good agreement between calculated and measured peak intensities, which is the main reason why this crystal structure was ruled out by Hoshino *et al.* (2003 ▸).

On the other hand, the peak positions and extinctions in our reciprocal space map provide evidence that this *C*2/*c* structure must exist. To resolve this contradiction, we assume varying molecular arrangements within the unit cell from crystallite to crystallite in the 2D powder of the CuPc thin films, which results in diffraction patterns with peak intensities that cannot be easily calculated. Since there is no ‘best’ configuration, we discuss possible configurations due to the excluded-volume considerations. There are 2^3^ = 8 main configurations (Fig. 4 ▸), two for each of the symmetry axes (yaw, pitch and roll). Coherent regions without molecular overlap appear in Fig. 4 ▸. Configurations within a coherent region are close to each other in the configuration matrix, *i.e.* assigned to neighboring matrix elements. So, continuous transitions from one configuration to another are possible.

Further calculations revealed that the molecules can glide past each other without penetrating the excluded volume, but also distinct areas appear in Fig. 4 ▸ meaning that not all configurations can be reached by a continuous transition. Such transitions would require an expansion followed by a contraction of the unit cell. Selected configurations are presented in Fig. S5 of the supporting information. The hydrogen atoms of neighboring molecules are interlocked. Furthermore, we found that the pitch angle cannot become 0° due to the limited space in the vertical direction. The same applies to the yaw angle due to the limited space in the horizontal direction. The roll angle stays well below 45° such that only the (×)-configuration is possible, which was denominated in this manner by Hoshino *et al.* (2003 ▸) in contrast to the (+)-configuration due to the orientation of the four benzene rings of CuPc with respect to the substrate surface.

### 4.4. Morphology   

**Figure 5:**
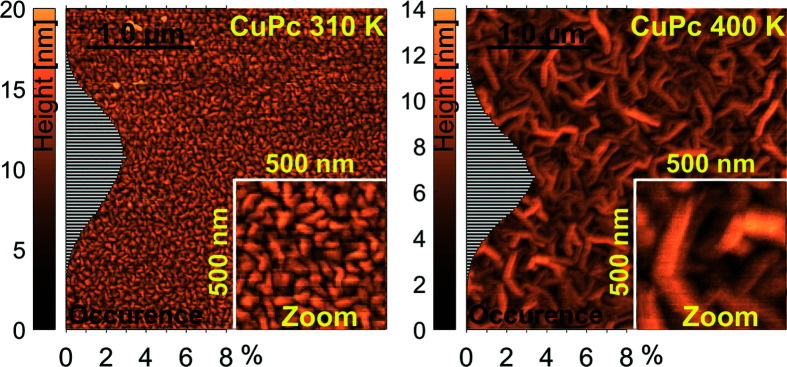
AFM: 3 × 3 µm images of 200 Å CuPc thin films grown at 2 Å min^−1^ and two different substrate temperatures, 310 and 400 K. The distribution of heights is shown as histograms on the left side of each image together with the color bar.

Regarding the AFM images in Fig. 5 ▸ and the analysis results in Table 1 ▸, we see that elevating the substrate temperature leads to two remarkable effects on the thin-film growth. Firstly, larger crystallites and larger islands form while the island density decreases by a factor of 10, and secondly, the CuPc film becomes smoother. The distinction between islands and crystallites is important, since the islands are larger than the size of coherent scattering domains, leading to the conclusion that each island consists of several crystallites. Despite all of the differences between 310 and 400 K, the worm-like shape of the islands is preserved at both substrate temperatures, as the insets in Fig. 5 ▸ show. Before we proceed, we note that our thin films exhibit a polycrystalline 2D-powder structure. The crystallites are more or less randomly oriented in the horizontal direction but are well ordered in the vertical direction. The layered ordering in the vertical direction is corroborated by the pronounced Bragg peaks in the XRR profiles in Fig. 2 ▸ and will be discussed in the following.

### 4.5. Layer coverages   

**Figure 6:**
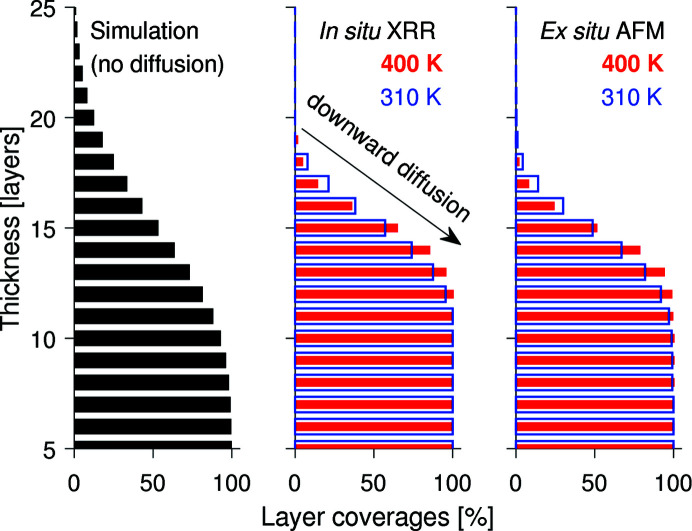
Experimentally determined layer coverages from *in situ* XRR and *ex situ* AFM of 200 Å CuPc thin films grown at 2 Å min^−1^ and two different substrate temperatures, 310 and 400 K, and comparison with a simulated layer coverage resulting from random deposition without diffusion or any kind of interaction.

Fig. 6 ▸ presents the simulated layer coverages compared with the coverages determined from *in situ* XRR and *ex situ* AFM. In the case of an absolutely smooth film, the first 15 layers would be completely filled and all further layers would be empty. Without any kind of molecular diffusion or interaction, the distribution of molecules to the distinct layers would look like the simulated layer coverages in the left part of Fig. 6 ▸. Missing matter from the lower 15 layers adds to the layers above, leading to a pronounced roughness. The heights are Poisson distributed and the roughness σ_RMS_ amounts to 13.1 Å × (15)^1/2^ ≃ 5.1 nm, which is much larger than the experimentally determined values summarized in Table 1 ▸. We conclude from this comparison that molecular downward diffusion occurred during the experiment. The evaluation of the XRR data resulted in 11–12 completely filled and seven partially filled layers, both at 310 and at 400 K, though the coverages of the lower layers are larger at 400 K than at 310 K, indicating that heat facilitates the downward diffusion. A similar trend is seen when regarding the layer coverages extracted from AFM. The difference between σ_RMS_ determined from AFM and XRR is only 1–2 Å and validates the applicability of both techniques in this study. Owing to the small change between *in situ* XRR and *ex situ* AFM, we conclude that the diffusion mainly took place during the growth and no major post-growth effects occurred.

## 5. Discussion   

Here, we compare our results for CuPc with prior studies on other small organic molecules. We found CuPc crystals growing in stacks along the short *b* axis parallel to the substrate surface. The thin films became smoother and the lateral sizes of the CuPc crystallites increase at elevated substrate temperatures as a result of improved molecular diffusion and faster growth along the substrate. Similar results were also found for perfluorinated copper phthalocyanine (Ossó *et al.*, 2002 ▸), but the preferential growth direction changes due to the fluorination to along the *a* axis (Jiang *et al.*, 2017 ▸).

Whether organic thin films become rougher or smoother on heated substrates is dependent on many parameters, such as the deposition rate (Storzer *et al.*, 2017 ▸; Farahzadi *et al.*, 2010 ▸) and the chemical structure (Belova *et al.*, 2018 ▸; Reisz *et al.*, 2017 ▸). Furthermore, the roughness depends on the film thickness and the choice of substrate (Dürr *et al.*, 2003 ▸; Kowarik *et al.*, 2006 ▸; Yim & Jones, 2006 ▸; Hong *et al.*, 2008 ▸; Zhang *et al.*, 2009 ▸, 2011 ▸; Kim *et al.*, 2010 ▸; Hinderhofer *et al.*, 2011 ▸; Obaidulla & Giri, 2015 ▸; Nahm & Engstrom, 2016 ▸; Brillante *et al.*, 2017 ▸; Chiodini, Straub *et al.*, 2020 ▸). Some molecules such as pentacene form smoother thin films at elevated substrate temperatures (Yagi *et al.*, 2004 ▸).

Studies on other molecules such as buckminsterfullerene, C_60_, report the opposite behavior of film roughening during growth on heated substrates (Bommel *et al.*, 2015 ▸), which could be due to different molecular shapes and interaction energies. The spherical shape of C_60_ molecules does not support a special growth direction. Thermal annealing experiments addressed the molecular reorganization after growth (Hinderhofer *et al.*, 2012 ▸; Duva *et al.*, 2019 ▸; Chiodini, D’Avino *et al.*, 2020 ▸), which influences the roughness as well. The increasing island sizes at decreasing island densities as a result of heated substrates during growth are confirmed by a previous study on CuPc (Jungyoon *et al.*, 2003 ▸; Padma *et al.*, 2016 ▸) and were also found for another molecule named para-hexaphenyl (6P) (Frank & Winkler, 2008 ▸). Finally, the assumption that different polymorphs and molecular orientations coexist in CuPc thin films is justified and corroborated by the reported continuous transitions between different CuPc polymorphs (Heutz *et al.*, 2000 ▸; Berger *et al.*, 2000 ▸).

## 6. Summary and conclusions   

This study demonstrates that both α polymorphs of CuPc, the *C*2/*c* and the *P* 1 structures, coexist in vacuum-deposited CuPc thin films on native silicon oxide. Many possible molecular configurations within the *C*2/*c* structure were found by systematic excluded-volume considerations. The AFM images show a 2D powder with randomly oriented islands consisting of several smaller crystallites. It is likely that the molecular arrangement within the unit cell differs from crystallite to crystallite, leading to a superimposed diffraction pattern whereby the peak intensities cannot be easily calculated.

Furthermore, the influence of substrate temperature on the thin-film morphology was investigated. An elevated substrate temperature of 400 K during growth leads to laterally larger islands and larger crystallites while the worm-like shape of islands is preserved. A remarkable result, however, is the fact that the film grown at 400 K became smoother than that grown at 310 K. This smoothing requires a molecular downward diffusion towards the substrate, as we demonstrated by comparing the layer coverages from XRR and AFM with a simulated random deposition of non-diffusion particles. Reciprocal space mapping showed that CuPc crystals grow in stacks along the substrate surface.

We conclude from the elongated shape of the islands that CuPc crystals grow faster along the stacking direction than perpendicular to it. This effect may be supported by the faster molecular diffusion at elevated substrate temperatures, resulting in a pronounced growth along the substrate surface and hence in a smoother thin film. Knowing the crystal structure, the morphology and how the growth can be influenced opens doors for further investigations such as charge transfer, mixing with other molecules and technical applications. 

## Supplementary Material

Additional plots and background information on the data evaluation. DOI: 10.1107/S1600576720015472/oc5004sup1.pdf

## Figures and Tables

**Table 1:** Size of coherent scattering crystallites *d*_coh_, island density ρ and root mean square roughness σ_RMS_ from XRR and AFM of 200 Å thin CuPc films grown at two different substrate temperatures (*T*_sub_)

*T*_sub_ (K)	*d*_coh_ (nm)	ρ (µm^2^)	σ_RMS_, XRR (nm)	σ_RMS_, AFM (nm)
310	21	400	2.9	2.7
400	31	40	1.8	1.7
