# Supplementary material for: Polymorphism and structure formation in copper phthalocyanine thin films
Source: J Appl Crystallogr. 2021 Feb 1;54(Pt 1):203–10. doi: 10.1107/S1600576720015472 (PMC7941321; doi:10.1107/S1600576720015472)

JOURNAL OF APPLIED CRYSTALLOGRAPHY

Volume 54 (2021)

**Supporting information for article:**

# Polymorphism and structure formation in copper phthalocyanine thin films

**Berthold Reisz, Valentina Belova, Giuliano Duva, Clemens Zeiser, Martin Hodas, Jakub Hagara, Peter Šiffalovič, Linus Pithan, Takuya Hosokai, Alexander Hinderhofer, Alexander Gerlach and Frank Schreiber**

# Supporting Information

## Polymorphism and Structure Formation in Copper Phthalocyanine Thin Films

B. Reisz, V. Belova, G. Duva, C. Zeiser, L. Pithan, J. Hagara, M. Hodas,
P. Šiffalovič, T. Hosokai, A. Hinderhofer, A. Gerlach, F. Schreiber

## 1. Layer Model

The X-ray reflectivity (XRR) profiles of CuPc grown at 310 K and at 400 K were fitted by a multi-layer model using the software GenX.[1] The number of completely filled layers was automatically fitted by the software. The number of incomplete (partially filled) layers was increased manually until the final value in the Figure-of-Merrit (FOM), which is a measure for the goodness of the fit, was less than 0.1. Beyond 7 incomplete layers, the FOM-value did not drop further and the number of completely filled layers approached an integer value of 11 at 310 K and 12 at 400 K, see Fig. **S1**.

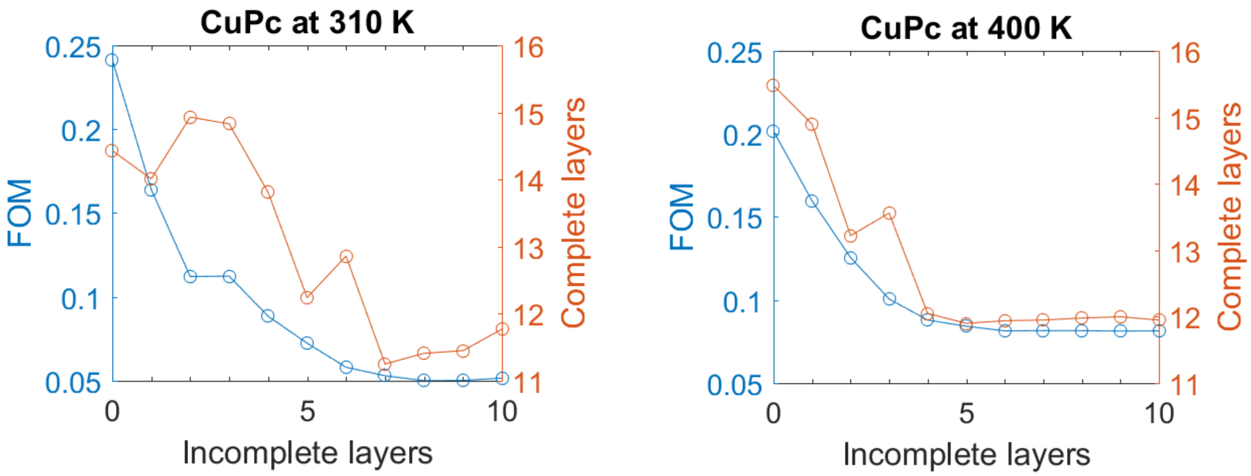


**Figure S1:** Final value resulting from the Figure-of-Merrit (FOM) and the fitted number of completely filled layers as a function of the number of incomplete (partially filled) layers.

---

[1] *M. Björck and G. Andersson, "GenX: An extensible X-ray reflectivity refinement program utilizing differential evolution", J. Appl. Cryst.* ***40*** *(2007), pp. 1174–1178*

## 2. Reciprocal Space Maps (Q-Maps)

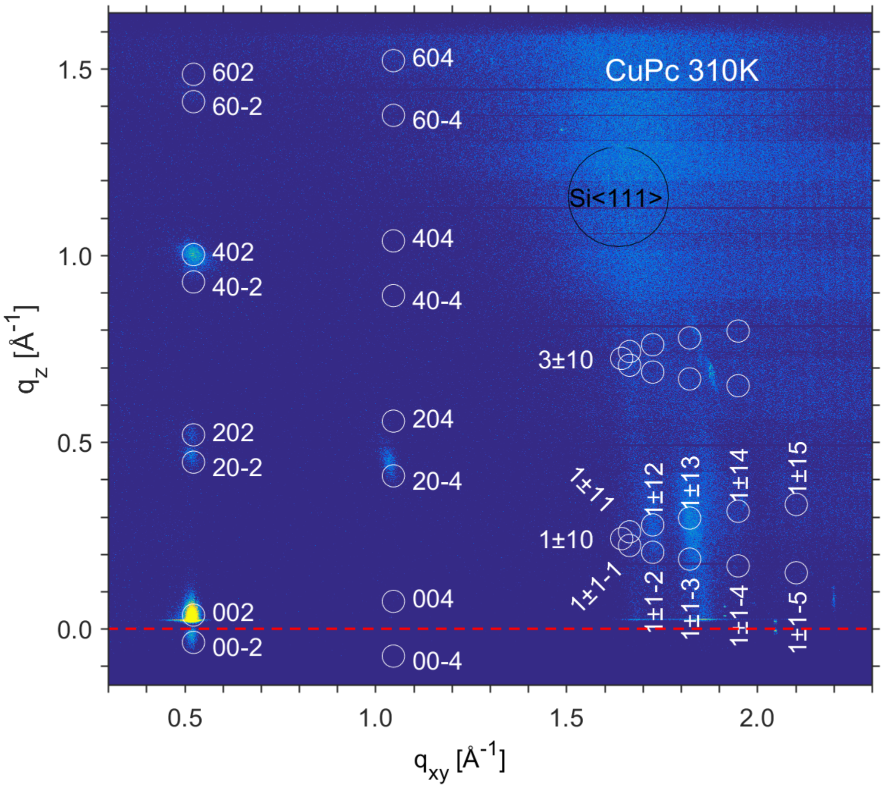


**Figure S2:**

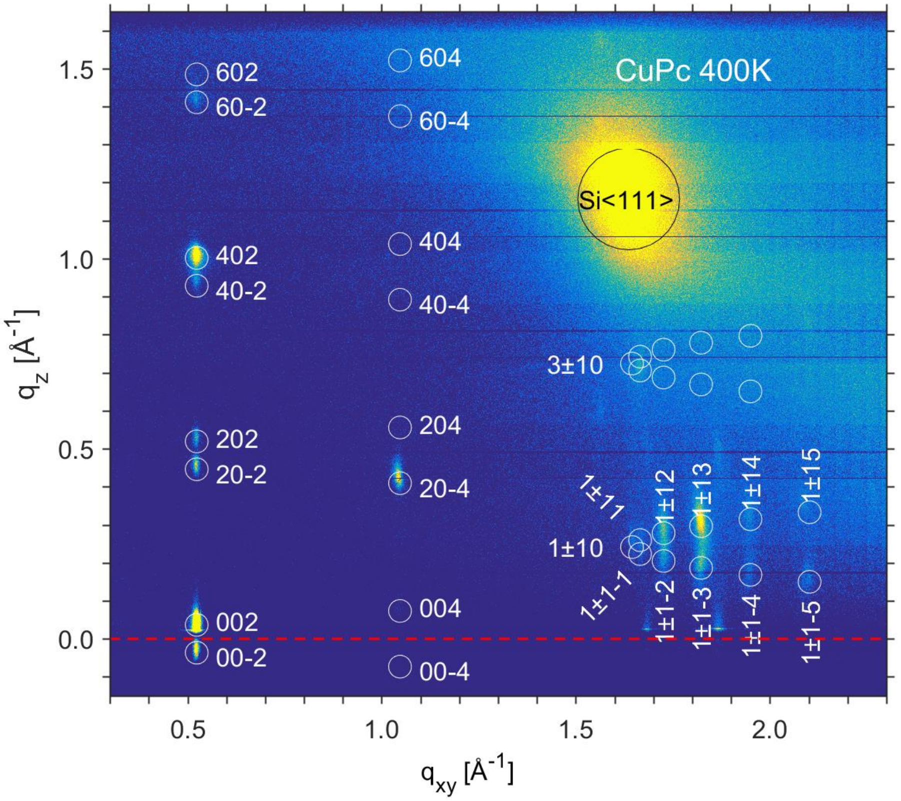


**Figure S3:**

# 3. Unit Cell and Crystal Structure

Table **T1** provides an overview of the unit cell parameters and the space groups of various polymorphic CuPc-crystal structures. The last column presents the CuPc thin film structure found in this study.

| | β-CuPc | α-CuPc | β-CuPc | α-CuPc | α-CuPc |
|---|---|---|---|---|---|
| | Robertson 1935 | Ashida et al. 1966 | Brown 1968 | Hoshino et al. 2003 | Thin film 2020 (new) |
| a [Å] | 19.6 | 25.29 | 19.407 | 12.886(2) | 26.1 |
| b [Å] | 4.79 | 3.79 | 4.790 | 3.769(3) | 3.82 |
| c [Å] | 14.6 | 23.29 | 14.628 | 12.061(3) | 24.0 |
| α | 90 | 90 | 90 | 96.22(7) | 90.0 |
| β | 120.6 | 90.4 | 120.93 | 90.62(4) | 94.0 |
| γ | 90 | 90 | 90 | 90.32(8) | 90.0 |
| V [Å³] | 1179.82 | 2292.6546 | 1166.44 | 582.3(5) | 2397 |
| Z | 2 | 4 | 2 | 1 | 4 |
| Space Group | $P\,2_1/a$ | $C2/c$ | $P\,2_1/a$ | $P\bar{1}$ | $C2/c$ |

**Table T1:** Unit cells of CuPc-polymorphs

---

*J. M. Robertson, "An X-ray study of the structure of the phthalocyanines. Part I. the metal free, nickel, copper, and platinum compounds," J. Chem. Soc.* ***136*** *(1935), pp. 615–621*

*M. Ashida, N. Uyeda, and E. Suito, "Unit cell metastable-form constants of various phthalocyanines," Bull. Chem. Soc. Jpn.* ***39*** *(1966), pp. 2616–2624*

*C. Brown, "Crystal structure of α-copper phthalocyanine," J. Chem. Soc.* ***A*** *(1968), pp. 2488 – 2493*

*A. Hoshino, Y. Takenaka, and H. Miyaji, "Redetermination of the crystal structure of α-copper phthalocyanine grown on KCl," Acta Cryst.* ***B59*** *(2003), pp. 393–403, 2003.*

Table **T2** provides a list of extinction/reflection conditions for the specific space group $C2/c$. A derivation of the extinction conditions is deduced from kinematic scattering theory. For more background information the reader is referred to the International Tables for Crystallography A.[2] Relative peak intensities were calculated by $I = \left|f\left(\vec{G}_{hkl}\right)\right|^2$. The atomic form factors $f_j(\vec{G}_{hkl})$ were taken from the International Tables for Crystallography C.[3]

---

[2] *T. Hahn and A. Looijenga-Voss, "Space-group symmetry", International Tables for Crystallography,* ***Vol. A*** *(2006), Chapter 2.2.13. Reflection Conditions, pp. 29–32*

[3] *E. N. Maslen, A. Fox, and M. A. O'Keefe, "Mathematical, physical and chemical tables", International Tables for Crystallography,* ***Vol. C*** *(2006), Chapter 6.1.1. X-ray Scattering, pp. 578-580*

**Kinematic Scattering Factor:**

$$f(\vec{G}_{hkl}) = \sum_j f_j(\vec{G}_{hkl}) \cdot \exp(2\pi i \cdot \vec{G}_{hkl} \cdot \vec{r}_j) = 0$$

| **Symmetry Element** | **Extinct (*hkl*)-Reflections** |
|---|---|
| C-centering (in the a-b-plane) | (*hkl*)-reflections with *h+k* being odd |
| 2-fold screw axis parallel to the unique b-axis | (*0k0*)-reflections with *k* being odd |
| Gliding mirror plane along the c-axis | (*h0l*)-reflections with *l* being odd |

**Table T2:** Extinction conditions for the space group C2/c

In the following, the kinematic scattering factor $f$ will be calculated for two equivalent atoms ($j = 1{,}2$) of the same species obeying the symmetries of the space group $C2/c$:

**C-centering (in the a-b-plane):** (integral extinction at specific points in the reciprocal space)

$$\vec{r}_1 = [x, y, z] \qquad \vec{r}_2 = \left[x + \tfrac{1}{2}, y + \tfrac{1}{2}, z\right]$$

$$f = \exp(2\pi i \cdot (x + y + z)) \cdot \left(1 + exp\left(2\pi i\left(\tfrac{1}{2} \cdot h + \tfrac{1}{2} \cdot k\right)\right)\right)$$

The extinction condition $f = 0$ is satisfied if $h + k \neq 2n$ (odd, $n =$ integer number)

**2-fold screw axis along the unique b-axis:** (serial extinction along an axis in the reciprocal space)

$$\vec{r}_1 = [x, y, z] \qquad \vec{r}_2 = \left[-x, y + \tfrac{1}{2}, -z\right]$$

$$f = \exp(2\pi i \cdot (x + y + z)) \cdot \left(1 + exp\left(2\pi i\left(-2 \cdot h \cdot x + \tfrac{1}{2} \cdot k - 2 \cdot l \cdot z\right)\right)\right)$$

The extinction condition $f = 0$ is satisfied if h = 0 and l = 0 and $k \neq 2n$ (odd, $n =$ integer number)

**Gliding mirror plane along the c-axis:** (zonal extinction along a plane in the reciprocal space)

$$\vec{r}_1 = [x, y, z] \qquad \vec{r}_2 = \left[x, -y, z + \tfrac{1}{2}\right]$$

$$f = \exp(2\pi i \cdot (x + y + z)) \cdot \left(1 + exp\left(2\pi i\left(-2 \cdot k \cdot y + \tfrac{1}{2} \cdot l\right)\right)\right)$$

The extinction condition $f = 0$ is satisfied if k = 0 and $l \neq 2n$ (odd, $n =$ integer number)

# 4. Molecular Orientation and Degree of Overlap

The CuPc-molecule was systematically rotated around its symmetry axis (yaw, pitch and roll in this order) from -45° to +45° in steps of 1.0°, respectively. Each rotation started from an upright standing molecule aligned with the unit cell such that the roll axis was parallel to the b-axis and the pitch axis was parallel to the c-axis. The other three molecules within each unit cell were rotated synchronously according to the symmetry operations of the space group C2/c.

The overlap $\delta_{ij}$ between each pair of atoms $i$ and $j$ was calculated by the difference between their distance $d_{ij}$ and the sum of their Van der Waals radii $d_{ij,\mathrm{VdW}}$. The overlap between molecules of neighboring unit cells was calculated by applying periodic boundary conditions to their atomic coordinates, i.e. copying protruding parts of each molecule to the opposite site of the unit cell. The sum of all $\delta_{ij}$ finally delivers the total degree of overlap in Angstrom for each configuration. Atomic pairs whose distance was larger than the sum of their Van der Waals radii and atomic pairs belonging to the same molecule were ignored in this sum.

Figure S2 shows dark spots at places in the configuration matrix whose total degree of overlap is less than 1.0 Å. Large coherent dark areas appear meaning that the molecules can glide past each other and reach new configurations without overlapping, but also distinct areas appear meaning that not all configurations can be reached by a continuous transition.

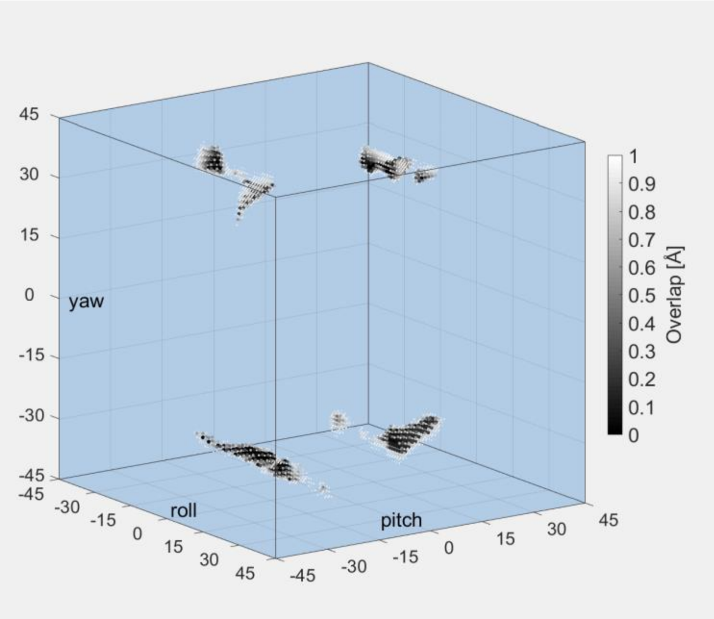


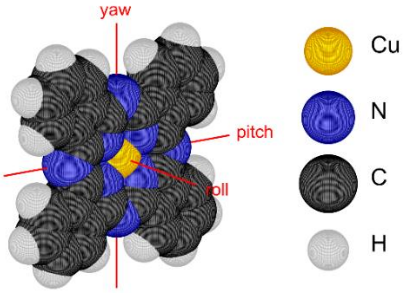


Van der Waals radii: [4]

| | |
|---|---|
| Cu | 1.40 Å |
| N | 1.55 Å |
| C | 1.70 Å |
| H | 1.20 Å |

**Figure S4:** $91 \times 91 \times 91$-matrix showing configurations as dark spots whose total degree of overlap between neighboring CuPc-molecules is less than 1.0 Å for the crystal structure: space group $C2/c$, a = 26.1 Å, b = 3.82 Å, c = 24.0 Å, β = 94.0°.

Total degree of overlap:

$$\delta = \sum_{i\neq j}\left(d_{ij,\mathrm{VdW}} - d_{ij}\right) \quad \text{if} \quad d_{ij} < d_{ij,\mathrm{VdW}} \quad \text{and } i \text{ and } j \text{ do not belong to the same molecule}$$

[4] A. Bondi, "Van der Waals volumes and radii," J. Phys. Chem. C **86** (1964) 441-451

# 5. Possible Configurations without Overlap

**Figure S5:**

A possible CuPc configuration without overlap (Space Group C2/c):

| | | |
|---|---|---|
| a = 26.(1) Å, | α = 90° | yaw = +33° |
| b =3.8(2) Å, | β = 94.(0)° | pitch = -21° |
| c = 24.(0) Å, | γ = 90° | roll = +3° |

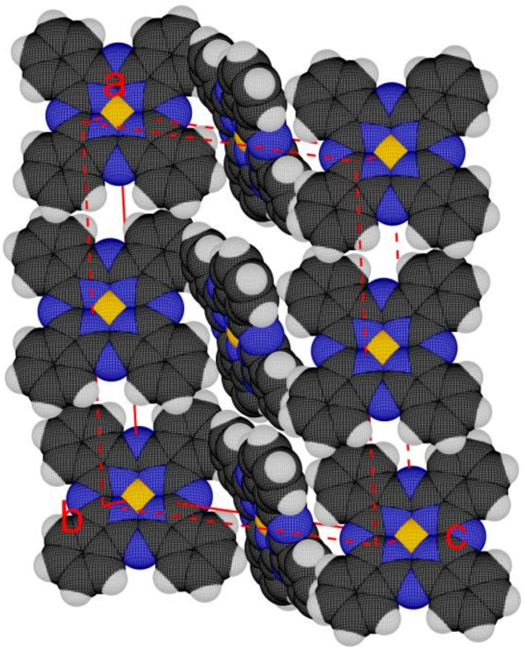


In order to demonstrate possible variations of the crystal structure, Fig. S6 presents two different configurations without overlap in a side view down the c-axis. The largest difference between both configurations is the pitch-angle, which exhibits a difference of 13°. The molecules are tilted forward in both cases. Note that for every pitch-angle, yaw- and roll-angle have to be adjusted in order to avoid molecular overlap. The hydrogen atoms of each molecule in the middle row have to fit into the gaps between the molecules of the upper and lower row, see Fig. 6.

A backward tilt would also be possible, but it does not represent a new configuration since a rotation of the entire crystal structure around the b-axis by 180° reproduces the same result when re-definition the unit cell box afterwards. A pitch-angle of 0° is not possible due to the limited space in vertical direction.

**Figure S6:**

View down the c-axis
for two different configurations.

| **Left:** | **Right:** |
|---|---|
| yaw = -34°, | yaw = -29° |
| pitch = -16°, | pitch = -29° |
| roll = -2°, | roll = -8° |

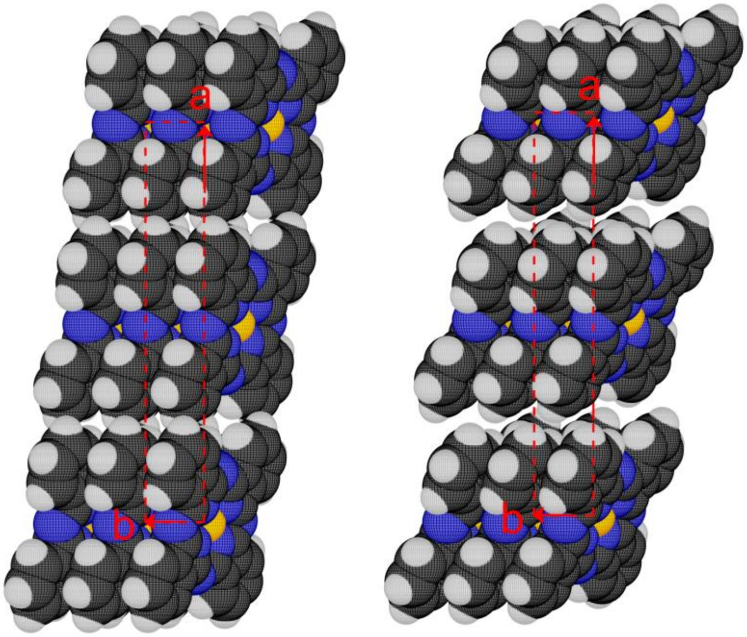

Supplement: Supplementary file 1 [file j-54-00203-sup1.pdf]
